# Role of the gut microbiome in mediating lactose intolerance symptoms

**DOI:** 10.1136/gutjnl-2020-323911

**Published:** 2021-03-18

**Authors:** M F Brandao Gois, Trishla Sinha, Johanne E Spreckels, Arnau Vich Vila, Laura A Bolte, Rinse K Weersma, Cisca Wijmenga, Jingyuan Fu, Alexandra Zhernakova, Alexander Kurilshikov

**Affiliations:** 1 Department of Genetics, University Medical Centre Groningen, Groningen, The Netherlands; 2 Department of Gastroenterology and Hepatology, University Medical Centre Groningen, Groningen, The Netherlands; 3 Department of Pediatrics, University Medical Centre Groningen, Groningen, The Netherlands

**Keywords:** diet, lactase, bifidobacteria, bacterial interactions, malabsorption

Misselwitz *et al* recently reviewed the multifactorial aspects of lactose intolerance (LI).^1^ Their work highlights the known effects of genetic makeup and dietary patterns in the occurrence of gastrointestinal symptoms in LI individuals. The authors define LI as the occurrence of gastrointestinal complaints on lactose exposure and discuss the wide variety of symptoms among LI patients.^1^ Regarding lactose metabolism, Misselwitz *et al* mention the influence of the gut microbiome, in particular that *Bifidobacterium* or other lactose-fermenting bacteria are reported to affect the levels of lactose in the gut. However, the impact of the gut microbiome on the occurrence of gut-related LI symptoms remains unclear.

We previously observed that the *Bifidobacterium* abundance in the adult human gut depends on the interaction between LI genetic variants and dairy intake.^2^ This observation complements other findings indicating a mutual relationship between the gut microbiome and host lactose metabolism.^3–8^ However, these earlier analyses did not consider the occurrence of gastrointestinal symptoms. This inspired us to investigate whether the interplay of dairy consumption and *Bifidobacterium* affects the occurrence of gastrointestinal complaints in LI individuals.

We analysed data on gut complaints, genetics, gut microbiome and diet in 959 participants of the Lifelines-DEEP Dutch population cohort. We classified LI genetic status based on the presence of the functional variant rs4988235 associated with LI in Caucasian populations, as described earlier,^2^ and we found the LI recessive genotype (G/G) in 81 individuals (8.4% of the cohort). We investigated microbiome composition using shotgun metagenomic sequencing of faecal samples^2^ and selected the relative abundance of the *Bifidobacterium* genus for our analysis. Dietary intake, including milk and other dairy products, was assessed using a food frequency questionnaire. Total dairy consumption, in grams per day, was retrieved using the Dutch Food Composition tables.^9^ Notably, there were no reports of complete avoidance of dairy consumption among patients with LI. Lastly, gut complaints were assessed via a 7-day questionnaire where participants were asked to rank (from 1 to 5) their daily level of gastrointestinal discomfort in six categories: ‘abdominal discomfort’, ‘bloating’, ‘burping’, ‘abdominal pain’, ‘flatulence’ and ‘nausea’. The sum of the mean daily scores from each category was used as the overall score of gastrointestinal complaints.

**Figure 1:**
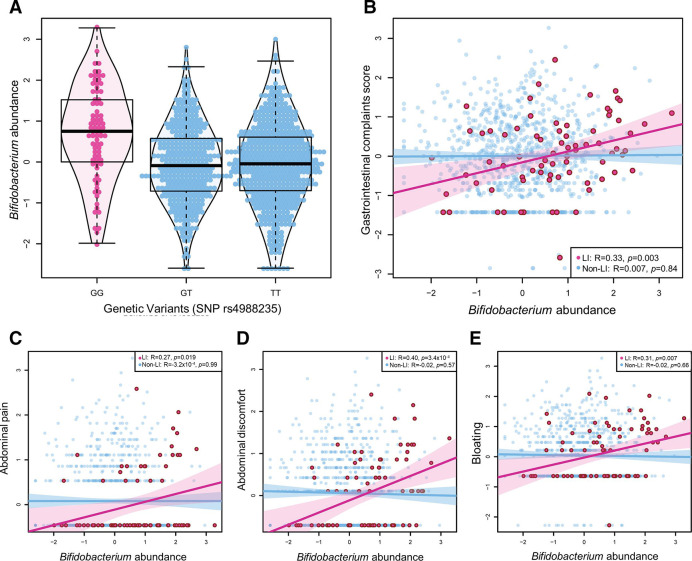
*Bifidobacterium* abundance and gut complaints in lactose intolerant (LI) individuals. (A) Higher *Bifidobacterium* abundance in individuals with the LI genotype (homozygous G/G) compared with the lactose tolerant genotypes (G/T and T/T) for SNP rs4988235. (B) In LI individuals, *Bifidobacterium* abundance is significantly associated with the total gastrointestinal complaints score (p=0.003). More specifically, *Bifidobacterium* abundance shows positive correlations with (C) abdominal pain, (D) gastrointestinal discomfort and (E) bloating. all numerical factors were subjected to rank-based inverse normal transformation.

**Figure 2:**
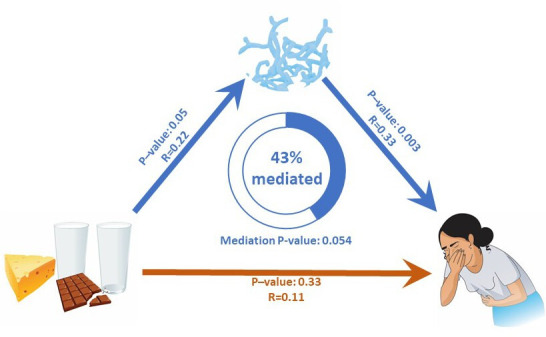
Analysis of the direct and indirect associations between milk intake, *Bifidobacterium* abundance and gut complaints. Statistically significant associations are shown with blue arrows. The non-significant correlation is shown by the red arrow. The presence of gut complaints related to the consumption of dairy products in LI individuals is largely mediated by *Bifidobacterium* abundance. LI, lactose intolerance.

As reported previously, an increased *Bifidobacterium* abundance was observed in LI individuals compared with non-LI individuals (*p*_Wilcox_=4.56×10^-9^) (figure 1A) and was positively correlated with dairy intake in the LI group (R=0.22, p=0.05) but not in the non-LI group (R=0.02, p=0.48). No significant difference in dairy consumption was found between the LI and non-LI groups (*p*_Wilcox_=0.31). In LI individuals, we observed a positive correlation between *Bifidobacterium* abundance and total gut complaints score (R=0.33, p=0.003) (figure 1B). Of the six specific gut complaints, *Bifidobacterium* abundance was positively correlated with abdominal pain, discomfort and bloating (figure 1C–E) but not with any other complaints (all p>0.26). We then performed a mediation analysis to address how dairy intake and *Bifidobacterium* abundance would influence the occurrence of symptoms in LI individuals. Interestingly, we found that the association between dairy intake and gastrointestinal complaints was partially mediated by *Bifidobacterium* abundance (Prop_mediated_=43%, p=0.054) (figure 2).

Our results provide evidence that specific gut symptoms, experienced by LI patients, might be the result of *Bifidobacterium* abundance in the gut, rather than a direct effect of lactose intake. This work supports initial reports where metabolic products of lactose-fermenting bacteria may be related to LI symptom occurrence.^7 8 10^
